# Graphene Quantum Dots-Based Electrochemical Biosensing Platform for Early Detection of Acute Myocardial Infarction

**DOI:** 10.3390/bios12020077

**Published:** 2022-01-28

**Authors:** Tanveer A. Tabish, Hasan Hayat, Aumber Abbas, Roger J. Narayan

**Affiliations:** 1 Department of Materials and London Centre for Nanotechnology, Imperial College London, London SW7 2AZ, UK; t.tabish@imperial.ac.uk; 2 College of Engineering, Swansea University, Wales SA1 8EN, UK; hasan.hayat@swansea.ac.uk; 3 School of Engineering, Newcastle University, Newcastle upon Tyne NE1 7RU, UK; a.abbas2@newcastle.ac.uk; 4 Joint Department of Biomedical Engineering, North Carolina and North Carolina State University, Raleigh, NC 27695-7907, USA

**Keywords:** myocardial infarction, graphene quantum dots, electrochemical, biosensing

## Abstract

Heart failure resulting from acute myocardial infarction (AMI) is an important global health problem. Treatments of heart failure and AMI have improved significantly over the past two decades; however, the available diagnostic tests only give limited insights into these heterogeneous conditions at a reversible stage and are not precise enough to evaluate the status of the tissue at high risk. Innovative diagnostic tools for more accurate, more reliable, and early diagnosis of AMI are urgently needed. A promising solution is the timely identification of prognostic biomarkers, which is crucial for patients with AMI, as myocardial dysfunction and infarction lead to more severe and irreversible changes in the cardiovascular system over time. The currently available biomarkers for AMI detection include cardiac troponin I (cTnI), cardiac troponin T (cTnT), myoglobin, lactate dehydrogenase, C-reactive protein, and creatine kinase and myoglobin. Most recently, electrochemical biosensing technologies coupled with graphene quantum dots (GQDs) have emerged as a promising platform for the identification of troponin and myoglobin. The results suggest that GQDs-integrated electrochemical biosensors can provide useful prognostic information about AMI at an early, reversible, and potentially curable stage. GQDs offer several advantages over other nanomaterials that are used for the electrochemical detection of AMI such as strong interactions between cTnI and GQDs, low biomarker consumption, and reusability of the electrode; graphene-modified electrodes demonstrate excellent electrochemical responses due to the conductive nature of graphene and other features of GQDs (e.g., high specific surface area, π–π interactions with the analyte, facile electron-transfer mechanisms, size-dependent optical features, interplay between bandgap and photoluminescence, electrochemical luminescence emission capability, biocompatibility, and ease of functionalization). Other advantages include the presence of functional groups such as hydroxyl, carboxyl, carbonyl, and epoxide groups, which enhance the solubility and dispersibility of GQDs in a wide variety of solvents and biological media. In this perspective article, we consider the emerging knowledge regarding the early detection of AMI using GQDs-based electrochemical sensors and address the potential role of this sensing technology which might lead to more efficient care of patients with AMI.

## 1. Introduction

Cardiovascular disease remains a leading cause of mortality and hospitalization worldwide. Despite significant advances in surgical interventions and pharmaceutical regimens, there are still inevitable risks associated with poor outcomes of many cardiovascular diseases such as acute myocardial infarction (AMI), thrombosis, angina, heart failure, restenosis, and myocardial reperfusion injury. AMI represents a leading cause of death among patients with cardiovascular diseases [1]. AMI occurs when one or more arteries that are responsible for the supply of blood to the heart muscle known as the myocardium become obstructed or blocked [2]. The narrowed or blocked arteries in the heart reduce or stop the blood supply to the myocardium, which in turn causes heart dysfunction. The persistent low blood supply results in low blood pressure and cardiac output, which leads to the occurrence of multiple organ dysfunction syndrome. AMI, as a complex and heterogeneous disease, is characterized by progressively debilitating and inevitability lethal biochemical and pathological processes [3]. This process is often diagnosed at a late irreversible stage; therefore, early stage detection of AMI is highly important. The early detection of AMI could facilitate rapid-response prevention and therapeutic interventions before the onset of heart dysfunction. An early therapeutic approach undoubtedly forestalls several enduring social and financial burdens. The identification of biomarkers is one way by which AMI can be diagnosed to both (a) detect disease onset and delay heart failure and in parallel (b) evaluate and track the therapeutic efficacy of drugs [4]. The global cardiac biomarker testing market is anticipated to exceed USD 13 billion by 2024 [5]. This market growth is primarily driven by high demand for biomarker tests for timely diagnosis as well as for drug development.

Many types of biomarkers have been identified for the detection of AMI, including but not limited to cardiac troponin [6], lactate dehydrogenase [7], myoglobin [8], creatine kinase and myoglobin [9], and C-reactive protein [10]. There has been significant research interest in discovering innovative biomarkers for the early detection of AMI. Troponin is a complex that contains three distinct proteins, including troponin T (30 kD molecular weight), troponin I (18 kD molecular weight), and troponin C (23 kD molecular weight), that control muscle contraction [11]. Troponin T and I are present in the heart. Cardiac troponin I (cTnI) has been demonstrated to be more specific and sensitive for the early detection of AMI even when it is present in ultralow concentrations [12]. The half-life of cTnI in the blood remains high for 4–7 days and comes down to normal within 7–10 days after the onset of myocardial damage. The concentration of cTnI in the patient’s blood increases to 0.3 ng/mL within 3–4 h of AMI [13]. In addition to cTnI, myoglobin is an important biomarker for the early identification of AMI. Myoglobin (17.8 kDa molecular weight) is a cytoplasmic oxygen-binding heme protein found in cardiac and skeletal muscles. Its typical concentration in the blood (30–90 ng/mL) rapidly rises within 1–3 h, peaks (∼200 ng/mL) within 6–12 h and comes down to normal within 24–36 h after the onset of AMI [14]. Although it is not as specific as cTnI, it has been demonstrated as an early biomarker over a short period (within 24 h) primarily due to its rapid release following AMI. Therefore, the precise, cost-effective, and convenient identification of predictive biomarkers is urgently needed to improve patient stratification.

**Figure 1:**
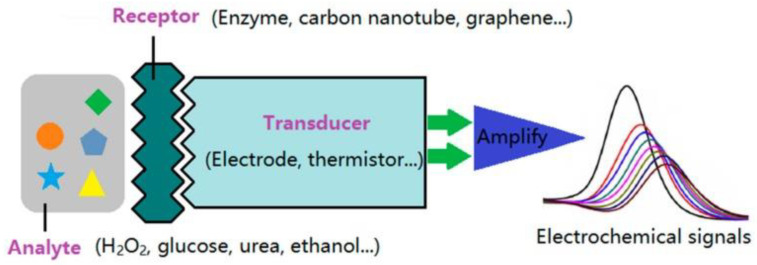
Schematic diagram of an electrochemical biosensor. Reprinted with permission from reference [22], copyright 2009 MDPI.

Biosensing methods used for the detection of cTnI and myoglobin include surface plasmon resonance [15], chemiluminescence [16], mass spectrometry [17], liquid chromatography [18], and fluorescence energy transfer [19]. Most of these methods are relatively specific and sensitive but costly and time consuming; furthermore, these methods require high-level complex instrumentation, which is not available in most healthcare settings. Biosensors are analytic tools that contain two components such as a biorecognition element (e.g., biomolecules such as enzymes, DNA, RNA, antibodies, and nucleic acids) and a transducer (e.g., an electrochemical, optical, acoustic, or thermal transducer) [20]. A transducer converts a particular biological event into a quantifiable and easily processible signal, which is proportional to the amount of target analyte under specific reaction conditions. Biosensing mechanisms involve a wide variety of approaches, including optical, electrical, and electrochemical mechanisms. The first form of electrochemical biosensor for the detection of blood glucose was reported by Clark et al. in 1962 [21]. Since then, several electrochemical biosensors have been developed and commercialized. An electrochemical sensor contains three electrodes: working, counter, and reference. The chemical reaction between the immobilized biomolecule and the analyte of interest takes place on the surface of the working electrode and produces or consumes ions/electrons. These ions/electrons create a potential from the reference electrode and generate a quantifiable signal. The integration of nanoparticles with electrochemical biosensing has remarkably upgraded the real-time, rapid, sensitive, specific, and reliable identification and quantification of cardiac biomarkers at very low cut-off concentrations for the early detection of cardiovascular diseases. The incorporation of nanomaterials alters the electrode surface and enhances the electrochemical activity of target analytes (even when they are present in very low concentrations) in comparison to the bare electrode [22]. A simple design of an electrochemical biosensor based on nanomaterials is shown schematically in Figure 1. Nanomaterials exhibit high adsorption of target analytes due to their high specific surface area and surface-to-volume ratio. Electrochemical biosensors integrated with nanomaterials offer several advantages over conventional biosensing platforms such as rapid and robust readout; selective, sensitive, label-free, and non-invasive detection; cost efficient; ease of fabrication; small amount of sample; and low background-to-noise ratio as well as user-friendly simple protocols and equipment. The key advantages of electrochemical biosensing systems in comparison to other methods such as chemiluminescence and fluorescence energy transfer are their robustness, flexibility of coating of electrodes, label-free detection of cardiac biomarkers based on electrochemical impedance spectroscopy (EIS), ease of miniaturization for point-of-care testing, real-time detection, multiplexed sensing, and exceptional detection limits at ultralow concentrations of biomolecules upon electrode/instrument optimization [23]. Furthermore, the screen and three-dimensional printing of electrodes is progressing rapidly. This printing strategy approach offers several benefits such as simple and cost-effective design, development, and high-scale production. Other techniques such as fluorescence energy transfer have some crucial limitations such as the complex treatment of fluorescent probes with surfactants, low sensitivity, and photobleaching and quenching, which restrict the sensitive and selective quantification of biomolecules in biological samples. Moreover, it is challenging to stock fluorescent samples for a long time [24]. In the case of chemiluminescence, the integration of chemiluminescence and electrochemical methods in the form of electrochemiluminescence is a promising approach for the ultrasensitive detection of biomolecules, such as DNA and micro RNA, that combines the advantages of both electrochemical and optical mechanisms [23].

A wide variety of nanomaterials have been considered for use in electrochemical biosensing such as nanoparticles [25], carbon nanotubes [26], graphene [27], polymeric nanostructures [28], and quantum dots (QDs) [29]. Graphene quantum dots (GQDs) are a promising new immobilizing agent for electrochemical biosensing of cardiac biomarkers owing to their unique features such as size-dependent optical, electrochemical, and electrochemiluminescent characteristics; quantum confinement; high surface area; chemical inertness; the existence of carboxyl and hydroxyl functional groups on their edges; ease of functionalization; variable bandgap energy; water solubility; and high biocompatibility [30]. The above-mentioned extraordinary optical and physiochemical features of GQDs make them an attractive candidate for electrochemical biosensing as compared to their counterparts such as pristine graphene, reduced graphene oxide, graphene oxide, and porous graphene nanosheets, as well as three-dimensional graphene foam and aerogels. GQDs are zero-dimensional nanocrystals of sp^2^-bonded carbon atoms that are arranged in a honeycomb structure; since the lateral dimensions of these materials are less than 100 nm, they exhibit an extremely high surface-to-volume ratio [30]. Graphene is considered to be a zero-bandgap material; zero-dimensional graphene in the form of GQDs exhibits excellent optical, catalytic, and electrochemical properties. GQDs have extensively been used in bioimaging [31], diagnostics [32], optoelectronics [33], photovoltaics [34], light-emitting diodes [35], drug delivery [36], and therapeutic applications (such as photodynamic therapy and a combination of photodynamic, photothermal, and chemical therapies) [37]. GQDs offer several advantages over conventional metallic or organic QDs such as photostability, water-solubility, and biocompatibility. The detailed synthesis of GQDs is beyond the scope of this minireview paper and has been discussed elsewhere [38,39].

The design and development of GQDs-integrated electrochemical biosensing for the early detection of AMI have emerged very recently and are currently on the research horizon. Although the use of GQDs-integrated electrochemical biosensing of cardiac biomarkers is at an early stage, some challenges currently remain unsolved. This minireview considers recent advances in electrochemical biosensing using GQDs for the early detection of AMI. Furthermore, the construction principles, antibody-free cost-efficient approaches for signal amplification, current challenges, and potentials of this technology are also discussed in this article.

## 2. GQDs-Based Electrochemical Biosensing for Early Diagnosis of Acute Myocardial Infarction

The output of an electrochemical sensor is quantified by amperometry voltammetry, electrochemical impedance spectroscopy, and impedimetry [40]. The incorporation of GQDs in electrochemical biosensing enhances the kinetics of electron transfer and redox reactions, which in turn significantly improves the sensitivity and specificity of target analyte detection. GQDs use redox-active biomolecules and facilitate direct electron transfer for the electrochemical detection of the target analyte. The exceptional electronic features of GQDs minimize the likelihood of passivation when they are used as an electrode or on the surface of an electrode in the form of a coating. In 2011, Zho et al. [41] reported the fabrication of electrochemical biosensors using GQDs-altered pyrolytic graphite electrodes; the attached ssDNA served as probes. Owing to the high conductivity of GQDs, the amended electrode exhibited an appropriate electrochemical response. Since then, GQDs have been used for the identification of a wide variety of biomarkers, but studies focusing on cardiac biomarkers are limited in number. Electrochemical biosensing coupled with GQDs for the detection of AMI is rapidly evolving.

**Figure 2:**
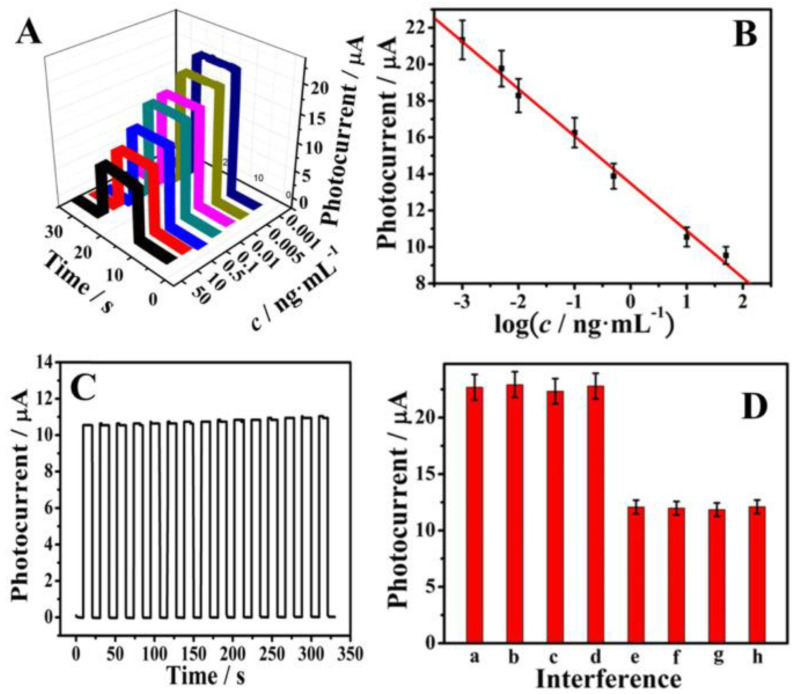
(**A**) Photocurrent response and (**B**) the logarithmic calibration curve for the sensor to detect cTnI in a concentration-dependent manner. (**C**) Photocurrent response of the sensor under 15 on/off illumination cycles for 320 s, concentration of cTnI = 10 ng/mL. (**D**) Selectivity of the sensor to detect cTnI: (a) Blank, (b) Blank + 100 ng/mL carcinoembryonic antigen, (c) Blank + 100 ng/mL squamous cell carcinoma antigen, (d) Blank + 100 ng/mL prostate-specific antigen, (e) 1 ng/mL cTnI, (f) 1 ng/mL cTnI + 100 ng/mL carcinoembryonic antigen, (g) 1 ng/mL cTnI + 100 ng/mL squamous cell carcinoma antigen, and (h) 1 ng/mL cTnI + 100 ng/mL prostate-specific antigen. Reprinted with permission from reference [44], copyright 2018 Elsevier.

In 2017, Bhatnagar et al. [42] reported the fabrication of cTnI antibody conjugated with GQDs and polyamidoamine (PAMAM) nanohybrid for the rapid electrochemical detection of cTnI in humans. This approach was associated with a sensitivity up to 109.23 μA cm^−2^/μg; a detection limit up to 20 fg/mL within 10 min was reported. In 2021, Mansuriya et al. [43] reported an electrochemical sensor containing a screen-printed gold electrode altered with GQDs and gold nanoparticles to identify cTnI for the early detection of AMI with a sensor sensitivity up to 6.81 µA cm^−2^ pg/mL. The sensor was prepared by applying GQDs and gold nanoparticles onto the gold electrode via drop coating, followed by the immobilization of the cTnI antibody onto the GQDs–gold nanoparticles nanocomposite. Detection of the analyte in human serum samples was demonstrated with detection limits of 0.1 and 0.5 pg/mL for buffer and serum, respectively. This sensing system was not evaluated for multianalyte analysis. However, such functionalized sensing systems utilize both antigens and antibodies and have several limitations, including low recovery, low efficiency, reagent use, multistep handling, long period for investigation, loading of antibodies, antibody fabrication costs, and poor stability and performance under extreme temperatures. The specificity of GQDs-functionalized electrodes without using antibodies has also emerged as a challenge for the real-time detection of biomolecules. Therefore, functionalized electrodes using doping agents without the use of antibodies could be exploited to increase the sensitivity, cost efficiency, and stability of the sensor. In response to this, several research groups have used nitrogen and sulfur as doping agents to control the photoelectric features of GQDs and further enhance their specificity towards the target analytes. The doping of nitrogen and sulfur has the ability to efficiently modify the electronic configuration of GQDs, which in turn can increase the kinetics of electron transport. For instance, Fan et al. [44] reported a label-free photoelectrochemical sensor containing nitrogen- and sulfur-doped GQDs, which were attached to a Zn_2_SnO_4_ cube ITO electrode both to accelerate electronic transfer and to enhance the photo-to-current conversion efficiency. This sensing platform was used to detect cTnI with a concentration range from 0.001 ng/mL to 50 ng/mL and with a detection limit of 0.3 pg/mL. Figure 2 illustrates the electrochemical response of nitrogen- and sulfur-doped GQDs conjugated with cubic Zn_2_SnO_4_. Figure 2A presents the photocurrent curves with increasing concentrations of cTnI which indicates the sensitivity of the as-prepared sensor for the detection of cTnI. Figure 2B shows the reduced linearity of the photocurrent with cTnI concentration (0.001 ng/mL to 50 ng/mL). Figure 2C confirms the photocurrent reaction of the sensor that was modified with 10 ng/mL cTnI under 15 cycles of illumination. Figure 2D shows the selectivity of the sensor for cTnI detection (at 1 ng/mL) in the presence of 100 ng/mL of carcinoembryonic, squamous cell carcinoma, and prostate-specific antigens. The results show that the GQDs-based sensor allows excellent specificity and sensitivity towards cTnI.

**Figure 3:**
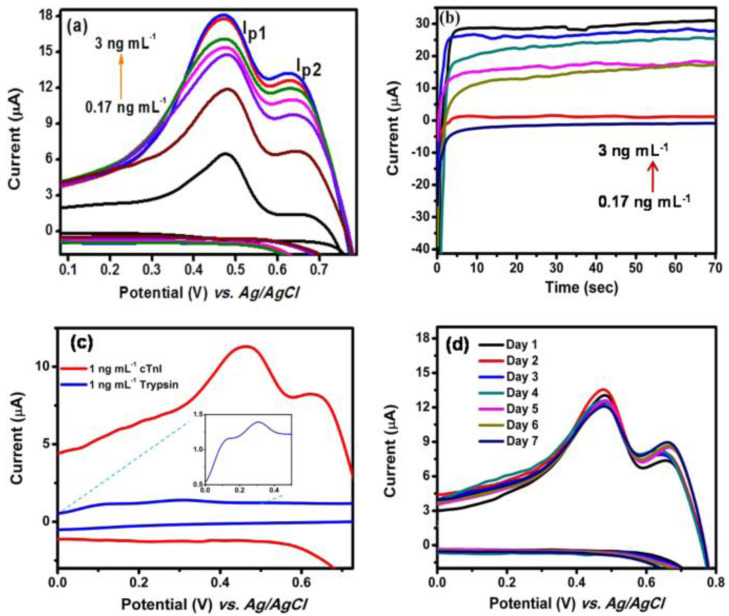
(**a**) Cyclic voltammogram of gold/functionalized-GQDs electrode for different concentrations of cTnI, (**b**) amperometric current response for different concentrations of cTnI at 460 mV, (**c**) cyclic voltammogram of gold/functionalized-GQDs electrode using trypsin, and (**d**) reproducibility (n = 3) study. Reprinted with permission from reference [45], copyright 2019 Nature Springer.

Antibody-free approaches based on biofunctionalized-GQDs-modified electrodes for electrochemical biosensors have recently gained attention. Antibody-free approaches could be cost efficient, stable, and involve straightforward protocols. In 2019, Lakshmanakumar et al. [45] used acetic acid-functionalized GQDs for the modification of a Au electrode for the electrochemical quantification of cTnI for the early detection of AMI with a sensitivity up to 3 ng/mL and a detection limit of 0.02 ng/mL. The coupling between the N-H group of cTnI and the COOH groups on the surface of the GQDs facilitated the sensitive detection of cTnI. Figure 3a shows the CV of the GQDs-modified Au electrode at different concentrations of cTnI. The oxidation peak currents were observed to rise with increased concentrations of cTnI (0.17 to 3.0 ng/mL). The detection limit of 0.02 ng/mL was associated with the conjugation of the functional groups of cTnI with the functional groups of the GQDs. Figure 3b displays a response time of 10 s. Figure 3c shows the anti-interfering capability of the sensor, where the CV reaction was documented in the presence of trypsin. Figure 3d validated the reproducibility of the sensor with a standard deviation of 3.1%. This study demonstrated an antibody-free approach for electrochemical biosensing of cTnI; additional validation of this approach is needed to facilitate use with human samples.

**Figure 4:**
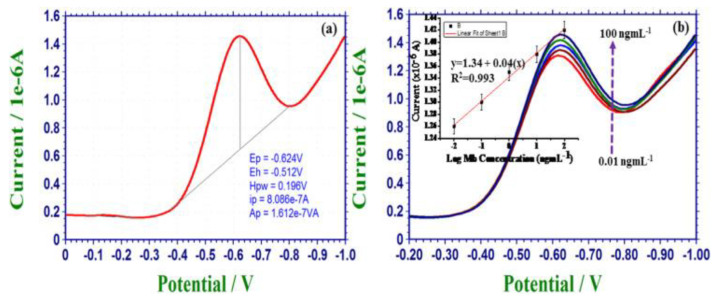
(**a**) DPV results for Ab/GQD/electrode-based electrochemical sensor in the presence of myoglobin and (**b**) DPV measurements as a function of myoglobin concentration; inset linearity graph. Reprinted with permission from reference [46], copyright 2016 Elsevier.

Tuteja et al. [46] described a label-free electrochemical sensor approach for the recognition of myoglobin by using GQDs on screen-printed electrode. The GQDs-modified electrode was further functionalized with anti-myoglobin antibodies in order to achieve the specificity of myoglobin. The charge-transfer resistance exhibited an increase from 0.20 to 0.31 kΩ in a linear manner over a concentration of 0.01–100 ng/mL myoglobin; a detection limit of 0.01 ng/mL was noted. The sensor showed specificity towards myoglobin when used with other proteins (e.g., CK-MB, hemoglobin, troponin I, avidin, and bovine serum albumin). The marked sensitivity of the sensor was attained by the immobilization of antibodies on the GQDs-modified electrode, which in turn accelerated the electron transfer of the sensor. Figure 4a shows the results of differential pulse voltammetry (DPV) analysis of the sensor in the presence of myoglobin. The peak at ~−0.62 V was reduced because of the decrease of the iron moiety existing in myoglobin. Figure 4b shows the decrease in current intensity with an increase in the concentration of myoglobin.

GQDs offer a wide range of advantages over other materials for the sensing of AMI, including the formation of strong interactions between cTnI and GQDs. In addition, graphene-modified electrodes exhibit excellent electrochemical responses due to the conductive nature of graphene and low levels of biomarker consumption; moreover, the reusability of the electrodes allows for several readings even after introducing new solutions [47]. In addition to these sensing-related characteristics, the attractive material properties of GQDs include a high specific surface area, the possibility of π–π interaction with the analyte, facile electron-transfer mechanisms, a tunable bandgap, biocompatibility, minimal toxicity, photoluminescence, electrochemical luminescence emission capability, effective redox properties, and ease of functionalization due to quantum-confinement effects. Another advantage of GQDs compared to other derivatives of graphene is the presence of functional groups such as hydroxyl, carboxyl, carbonyl, and epoxide groups; these functional groups improve the solubility and dispersibility of GQDs in a wide range of solvents and biological media [48]. The functional groups also enable the functionalization of GQDs with antibodies or other biomolecules for the selective detection of biomarkers at ultralow concentrations. When comparing the results of GQDs with those of other electrochemical biosensors for the detection of AMI (e.g., via cTnI) in the scientific literature, it can be observed that GQDs have shown better results than other nanoparticles in terms of signal amplification, electrochemical response, and storage of antibodies. For example, Ahammad et al. [49] described the electrodeposition of gold nanoparticles on indium tin oxide (ITO) electrodes and used these electrodes to detect cTnI by measuring open-circuit potential (OCP) values; they observed a linear dependence between OCP changes and cTnI levels over a range of concentrations from 1 to 100 ng/mL. In another study, Sandil et al. [50] studied the deposition of 3-aminopropyltriethoxysilane (APTES)-functionalized tungsten trioxide nanosheets on ITO electrodes via an electrophoretic deposition approach; they immobilized cardiac troponin I antibodies (anti-cTnI) on these structures. The structures enabled the detection of cTnI with a linear detection range of 0.1 to 100 ng mL^−1^; stability up to 6 weeks was observed. Moreover, the electrode was able to detect cTnI in spiked human serum.

The performance of other nanoparticles for the electrochemical detection of cardiac biomarkers has also been discussed elsewhere [51,52]. Table 1 summarizes the comparison of various classes of nanoparticles with GQDs-based electrochemical sensors for the detection of cTnI and myobglobin; this comparison indicates that GQDs-based electrochemical sensors have lower limits of detection compared to other types of nanoparticles.

## 3. Conclusions and Future Outlook

Accurate and early detection of AMI is challenging in clinical settings. Benchmark biomarkers such as cTnI and myoglobin represent the identification of early deterioration in myocardial function and hold promise to forecast the early make-up of AMI. These predictive biomarkers have been demonstrated to be useful in differentiating between diseased and non-diseased lesions. In the rapidly growing field of electrochemical biosensing, rapid and real-time monitoring of AMI is emerging as a possibility. A wide range of nanomaterials have been used for the detection of AMI biomarkers with promising results. Cost-efficient and ultrasensitive electrochemical biosensors made using nanomaterials such as gold, iron oxide, zinc oxide, manganese oxide, copper oxide, titanium oxide, and platinum nanoparticles as well as molybdenum disulfide nanosheets have shown promising results but limited applications for the detection of AMI. These nanoparticles show some limitations such as cost-inefficient synthesis routes, time-consuming synthesis routes, and low sensitivity in complex samples. More recently, combining GQDs with electrochemical biosensors has shown improved sensitivity, specificity, and selectivity for the detection of biomarkers associated with AMI. By virtue of the properties of GQDs such as their large specific surface area, surface-to-volume ratio, π–π stacking, biocompatibility, and minimal toxicity, the use of GQDs for electrochemical biosensing has the potential to advance the field of cardiac diagnostics. The key advantages offered by GQDs include a strong electrochemical response due to the conductive nature of graphene, the size-dependent optical features of GQDs, the capability for π–π bonding of GQDs with analytes, the availability of fast electron-transfer mechanisms due to the high electron mobility of graphene, and biocompatibility. This brief review considers the recent developments of different types of electrochemical biosensors along with their benefits and shortcomings. Several challenges need to be addressed to facilitate the clinical translation of GQDs-based electrochemical biosensors. In order to achieve high reliability and sensitivity of electrochemical biosensors, bioconjugation and biofunctionalization strategies, and the interplay between the size and shape of the biorecognition element and the choice of synthesis routes of GQDs and functionalization as well as multimarker identification, need to be optimized precisely. The concentrations of biomarkers could vary based on several factors, including sex, weight, and age. In addition, with globalization and a growing population, the application of biosensors must be cost efficient and straightforward to use in healthcare settings. Therefore, electrochemical biosensors should satisfy the standards of validation with statistical testing among different populations and high-risk groups at multiple locations. Previous studies reported on this topic have not specified the age, sex, or racial and ethnic backgrounds of the human samples used in the studies. Furthermore, high levels of troponin are also linked to other cardiac injuries; hence, capabilities such as speed, precision, and accuracy of detection of troponin associated with AMI need to be validated using human samples for clinical translation of the technology. A pronounced prospect exists for researchers to address these key technological gaps by carefully designing future studies in order to achieve high-quality diagnostic tools. These challenges enable us to design and develop wide-ranging and efficient biosensing platforms aimed at diagnosis as well as monitoring the response to a specific therapy. In conclusion, real-time and rapid monitoring and sensing regimes, if appropriately validated and used, offer the promise of mitigating the adverse outcomes associated with AMI.

## Figures and Tables

**Table 1:** Comparison of GQDs and other nanomaterials for the electrochemical detection of cTnI and myoglobin in terms of the detection method, detection range and detection limit.

Class of Nanomaterials	Biomarker	Sample Source	Electrochemical Method	Limit of Detection	Refs.
Gold nanoparticles	cTnI	Serum	Electrical impedance spectroscopy	1 pg mL^−1^	[53]
ZnO nanoparticles	cTnI	Serum	Electrical impedance spectroscopy	1 pg mL^−1^	[54]
Pt nanoparticles/G-carbon nanotubes	cTnI	Serum	Electrical impedance spectroscopy	1.0 pg mL^−1^	[55]
Gold nanodumbbells	cTnI	Serum	Differential pulse voltammetry	8.0 pg mL^−1^	[6]
Acetic acid functionalized GQDs	cTnI	Serum	Cyclic voltammetry and amperometry	0.02 ng mL^−1^	[45]
GQDs/polyamidoamine nanohybrid	cTnI	Serum	Cyclic voltammetry and Differential pulse voltammetry	20 fg mL^−1^	[42]
Gold nanoparticles	Myoglobin	Serum	Electrical impedance spectroscopy	2.7 ng mL^−1^	[56]
Multiwalled carbon nanotubes	Myoglobin	Serum	Cyclic voltammetry	0.171 pg mL^−1^	[57]
Gold nanoparticles @reduced graphene oxide	Myoglobin	Serum	Differential pulse voltammetry	0.67 ng mL^−1^	[58]
GQDs	Myoglobin	Serum	Electrical impedance spectroscopy	0.01 ng mL^−1^	[46]

## Data Availability

Not applicable.
